# Neurobehavioral effects of uremic toxin–indoxyl sulfate in the rat model

**DOI:** 10.1038/s41598-020-66421-y

**Published:** 2020-06-11

**Authors:** Malgorzata Karbowska, Justyna M. Hermanowicz, Anna Tankiewicz-Kwedlo, Bartlomiej Kalaska, Tomasz W. Kaminski, Krzysztof Nosek, Roza J. Wisniewska, Dariusz Pawlak

**Affiliations:** 1 Department of Pharmacodynamics, Medical University of Bialystok, Bialystok, Poland; 2 Department of Clinical Pharmacy, Medical University of Bialystok, Bialystok, Poland; 3 Department of Monitored Pharmacotherapy, Medical University of Bialystok, Bialystok, Poland; 4 Pittsburgh Heart, Lung and Blood Vascular Medicine Institute, University of Pittsburgh, Pittsburgh, PA 15260 USA; 5 Department of Pharmacology and Toxicology, University of Warmia and Mazury, Olsztyn, Poland; 6 Department of Pharmacology, Medical University of Bialystok, Bialystok, Poland

**Keywords:** Kidney diseases, Nephrology, Kidney diseases, Neurological disorders, Neurological disorders, Risk factors

## Abstract

Chronic kidney disease (CKD) is deemed to be a worldwide health concern connected with neurological manifestations. The etiology of central nervous system (CNS) disorders in CKD is still not fully understood, however particular attention is currently being paid to the impact of accumulated toxins. Indoxyl sulfate (IS) is one of the most potent uremic toxins. The purpose of the present study was to assess IS concentrations in the cerebellum, brainstem, cortex, hypothalamus, and striatum with hippocampus of rats chronically exposed to IS. To evaluate IS impact on neurochemical and behavioral alterations, we examined its influence on brain levels of norepinephrine, epinephrine, dopamine, serotonin and their metabolites, as well as changes in behavioral tests (open field test, elevated plus maze test, chimney test, T maze test, and splash test). Our results show the highest IS accumulation in the brainstem. IS leads to behavioral alterations involving apathetic behavior, increased stress sensitivity, and reduced locomotor and exploratory activity. Besides, IS contributes to the impairment of spatial memory and motor coordination. Furthermore, we observed reduced levels of norepinephrine, dopamine or serotonin, mainly in the brainstem. Our findings indicate that IS can be one of the crucial uremic factors responsible for altered mental status in CKD.

## Introduction

Chronic kidney disease (CKD) is deemed to be a growing worldwide health concern connected with neurological manifestations. The decline in estimated glomerular filtration rate (eGFR) correlates with an increase in the number of central nervous system (CNS) disorders like stroke, encephalopathy, and alteration in mental status involving confusion, disorientation, amnesia, disruptions in psychomotor skills, behavior changes, and other cognitive dysfunctions. The etiology of neurological complications during CKD is associated with neuronal injury or changes in cerebral neurotransmitters caused by cerebrovascular disease, secondary hyperparathyroidism, dialysis disequilibrium, electrolyte disturbances, and anemia^1–3^. However, much attention is currently focused on the accumulation of uremic toxins, which can lead to toxic-metabolic encephalopathy^4,5^.

Indoxyl sulfate (IS) is one of the most potent protein-bound uremic toxins that are well-known for nephrovascular toxicity^6,7^. However, IS can also be considered as a neurotoxin due to its ability to accumulate in the brain tissue, especially as a result of the dysfunction of organic anion transporter 3 (OAT 3) in the blood-brain barrier^8^. Importantly, IS can contribute to the disruption of CNS homeostasis and neuronal damage directly through an increase in oxidative stress and inflammation in glial cells^9^. Nevertheless, its aryl hydrocarbon receptor (AhR) agonistic property^10^, prothrombotic effect^11,12^, and IS-induced endothelial dysfunction^13^ can also indirectly lead to neuronal damage.

Available studies on animals with acute kidney injury or chronic renal insufficiency demonstrate both behavioral disturbances and alteration in neurotransmitters, particularly monoamine neurotransmitters. There are only a few data showing the cerebral level of IS^14–16^, but none of them reveal their sole influence on behavior and cerebral neurotransmitters. Therefore, the purpose of the present study was to assess IS concentrations in the cerebellum (B1), brainstem (B2), cortex (B3), hypothalamus (B4), and striatum with hippocampus (B5) of rats chronically exposed to IS. To evaluate IS impact on neurochemical and behavioral alterations, we examined its influence on brain levels of monoamines (norepinephrine – NE, epinephrine – E, dopamine – DA, serotonin – 5HT) and their metabolites (3,4-dihydroxyphenylacetic acid – DOPAC, 5-hydroxyindolacetic acid – 5HIAA), as well as changes in behavioral tests (open field test, elevated plus maze test, chimney test, T maze test, and splash test). The IS can contribute to neuronal damage through oxidative stress. For this purpose, the concentration of malondialdehyde (MDA) that is a lipid peroxidation marker was assessed.

## Results

### Cerebral level of IS

**Figure 1:**
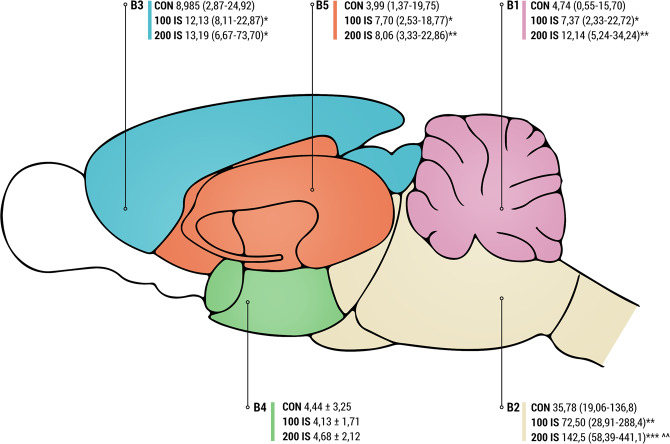
Level of IS in the cerebellum (B1), brainstem (B2), cerebral cortex (B3), hypothalamus (B4), and striatum with the hippocampus (B5) after chronic administration; n = 45. Data are presented the mean ± SD or as a median (full range) depending on their distribution. For comparison of IS concentration in B1, B2, B3, and B5 Kruskal-Wallis test has been used. For B4 ordinary ANOVA test has been used. To compare the differences between all the same dose in the different parts of the brain Kruskal-Wallis test has been used. The Fig. 1 was prepared by MK using Adobe Photoshop CC. B1 – cerebellum; B2 – brainstem; B3 – cerebral cortex; B4 – hypothalamus; B5 – striatum with hippocampus; CON – control group; 100 IS – group receiving IS in the dose of 100 mg/kg b.w./day; 200 IS – group receiving IS in the dose of 200 mg/kg b.w./day; IS – indoxyl sulfate; Results: B1 (P = 0.0006, Kruskal-Wallis statistic 14.90; *CON vs 100 IS P = 0.0288; **CON vs 200 IS P = 0.0003; Mann-Whitney test), B2 (P < 0.0001, Kruskal-Wallis statistic 20.58; **CON vs 100 IS P = 0.0093; ***CON vs 200 IS p < 0.0001; ^^100 IS vs 200 IS P = 0.0034; Mann-Whitney test), B3 (P = 0.012, Kruskal-Wallis statistic 9.409; *CON vs 100 IS P = 0.0152; *CON vs 200 IS P = 0.0411; Mann-Whitney test), B5 (P = 0.0166, Kruskal-Wallis statistic 8.200; *CON vs 100 IS P = 0.0192; **CON vs 200 IS P = 0.0055; Mann-Whitney test). The comparison of all the controls (P < 0.0001, Kruskal-Wallis statistic 44.5), comparison of all the 100 mg/kg doses (P < 0.0001, Kruskal-Wallis statistic 58.22), comparison of all the 200 mg/kg doses (P < 0.0001, Kruskal-Wallis statistic 54.06).

The regional level of IS in the brain is shown in Fig. 1. Chronic administration of IS led to a significant increase in IS concentration in every part of the brain except hypothalamus (B4 part). The highest IS concentrations were observed in the brainstem (B2 part). Additionally, significant differences between 100 IS and 200 IS groups were observed only in the brainstem IS concentrations (Fig. 1).

### General observation

The general characteristics of rats are shown in Table 1. There were no differences in the final body weight, food intake and water intake between control and experimental groups.

**Table 1:** IS impact on general characteristics.

	CON	100 IS	200 IS
Final body weight [g]	341.9 ± 14.1	348.9 ± 18.2 NS	351.2 ± 25.1 NS
Food intake [g/day]	28.52 ± 2.9	30.92 ± 1.5 NS	29.76 ± 3.6 NS
Water intake [ml/day]	37.90 ± 5.1	35.70 ± 1.6 NS	35.31 ± 2.8 NS

CON – control group; 100 IS – group receiving IS in the dose of 100 mg/kg b.w./day; 200 IS – group receiving IS in the dose of 200 mg/kg b.w./day; IS – indoxyl sulfate; NS – non-significant.

### Regional monoamines level

#### Regional NE, E levels and NE turnover

**Figure 2:**
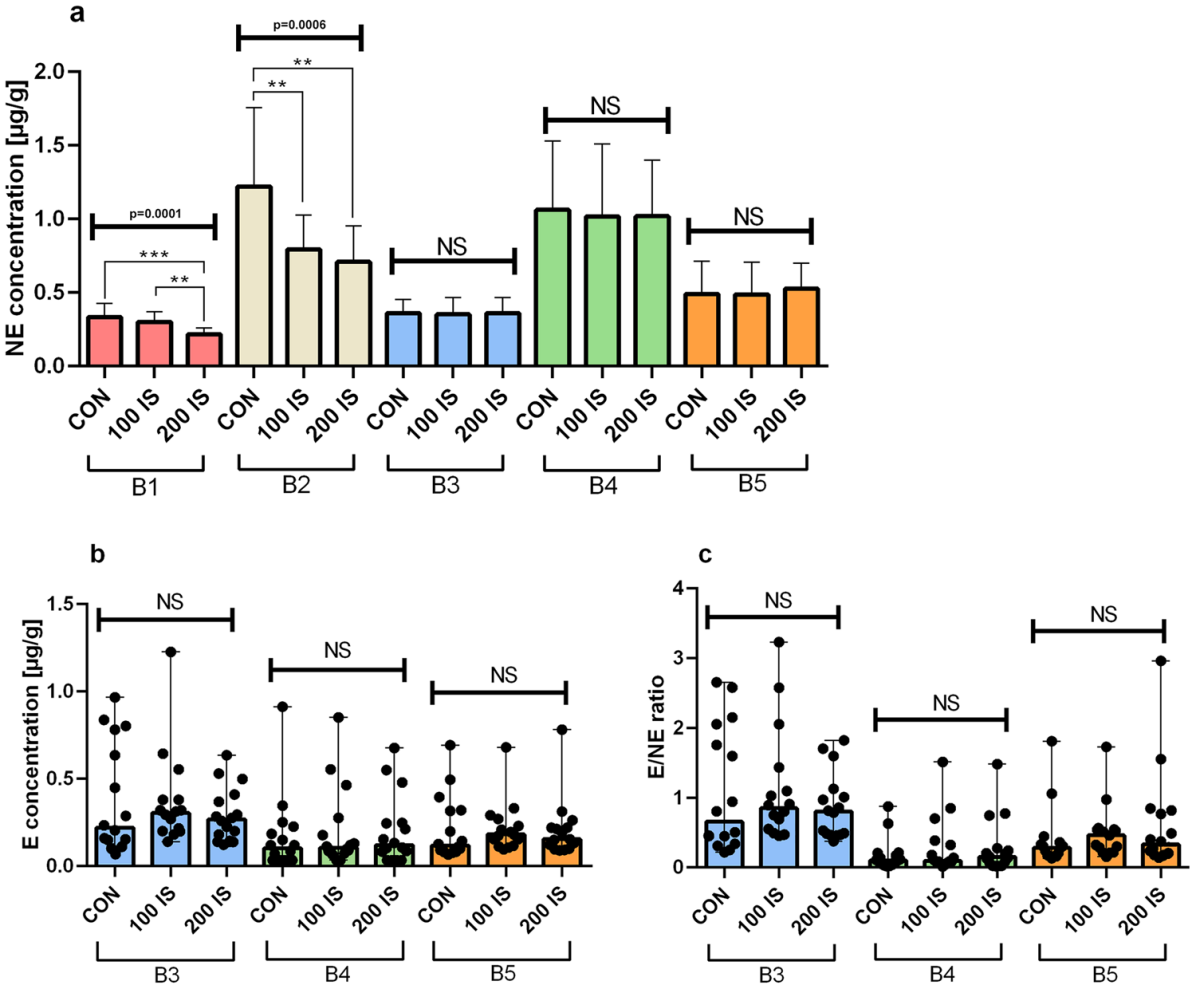
The effect of IS on regional NE (**a**), E levels (**b**) and NE turnover (**c**); n = 45. (**a**) Data are presented as the mean ± SD due to passed normality test (alpha=0.05). B1 – Anova – 0.0001 / F (DFn, DFd) = F (2, 42) = 11.83; Student t-test: ***CON vs 200 IS P = 0.0001; **100 IS vs 200 IS P = 0.0002. B2 – Anova – 0.0006 / F (DFn, DFd) = F (2, 42) = 9.074; Student t-test: **CON vs 100 IS P = 0.0068; **CON vs 200 IS P = 0.0017. All Controls Anova – 0.0001 / F (DFn, DFd) = F (4, 70) = 24.32. All 100 IS Anova – 0.0001 / F (DFn, DFd) = F (4, 70) = 19.92. All 200 IS Anova – 0.0001 / F (DFn, DFd) = F (4, 70) = 27.89. (**b**) Data are presented as a median (full range) due to unmeet normal distribution condition (alpha = 0.05). Data were analyzed using Kruskal-Wallis test. All Controls Kruskal-Wallis test – 0.0193 / Kruskal-Wallis statistic 7.897. All 100 IS Kruskal-Wallis test– 0.0025 / Kruskal-Wallis statistic 11.95. All 200 IS Kruskal-Wallis test – 0.0298 / Kruskal-Wallis statistic 7.024. (**c**) Data are presented as a median (full range) due to unmeet normal distribution condition (alpha=0.05). Data were analyzed using Kruskal-Wallis test. All Controls Kruskal-Wallis test – <0.001 / Kruskal-Wallis statistic 25.25. All 100 IS Kruskal-Wallis test– <0.0001 / Kruskal-Wallis statistic 17.94. All 200 IS Kruskal-Wallis test – 0.0003 / Kruskal-Wallis statistic 16.37. B1 – cerebellum; B2 – brainstem; B3 – cerebral cortex; B4 – hypothalamus; B5 – striatum with hippocampus; NE – norepinephrine; E – epinephrine; E/NE ratio – NE turnover; CON – control group; 100 IS – group receiving IS in the dose of 100 mg/kg b.w./day; 200 IS – group receiving IS in the dose of 200 mg/kg b.w./day.

Chronic exposure to IS resulted in decreased NE concentrations in the cerebellum (B1) (P = 0.0001, 200 IS) and brainstem (P = 0.0068, 100 IS and P = 0.0017, 200 IS) (Fig. 2a). The brain concentrations of E in each examined region did not differ between the control and IS groups (Fig. 2b). As shown in Fig. 2c, E/NE ratio presented as a measure of NE turnover also did not differ between control and experimental groups. E levels in the B1 and B2 parts of the brain were below the limit of detection.

#### Regional DA, DOPAC levels and DA turnover

**Figure 3:**
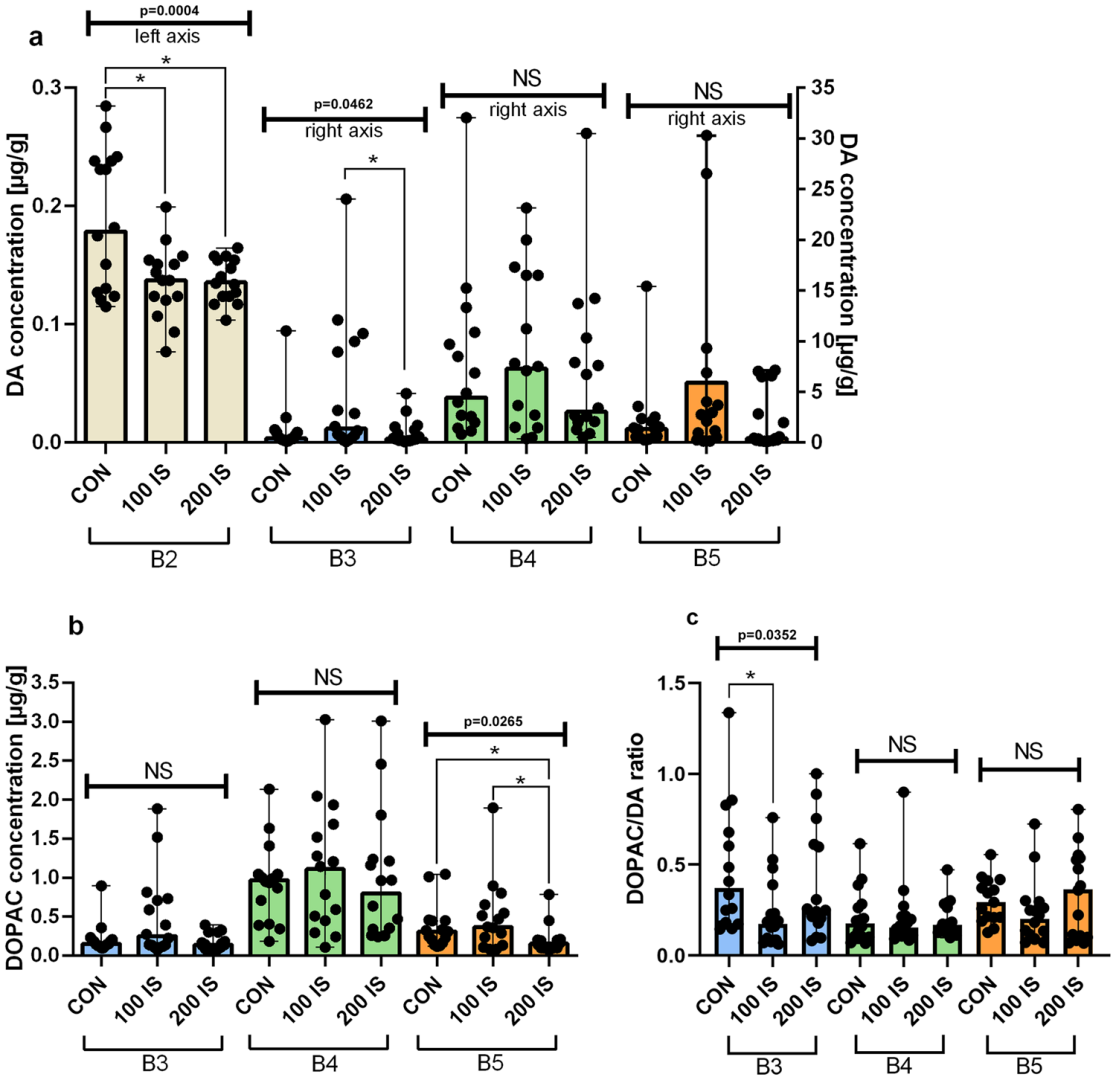
The effect of IS on regional DA (**a**), DOPAC levels (**b**), and DA turnover (**c**); n = 45. (**a**) Data are presented as a median (full range) due to unmeet normal distribution condition (alpha=0.05). For comparison of IS concentration in B3, B4, B5 Kruskal-Wallis test has been used. For B2 ordinary ANOVA test has been used. To compare the differences between all the same dose subgroups in the different parts of the brain Kruskal-Wallis test has been used. B2 - Anova – 0.0004 / F (DFn, DFd) = F (2, 42) = 8.995; Mann-Whitney test: *CON vs 100 IS P = 0.0244; *CON vs 200 IS P = 0.0304. B3 - Kruskal-Wallis test – 0.0462 / Kruskal-Wallis statistic 6.151; Mann-Whitney test: *100 IS vs 200 IS P = 0.038. All Controls Kruskal-Wallis test – <0.0001 / Kruskal-Wallis statistic 41.08. All 100 IS Kruskal-Wallis test– <0.0001 / Kruskal-Wallis statistic 29.81. All 200 IS Kruskal-Wallis test – <0.0001 / Kruskal-Wallis statistic 36.97. (**b**) Data are presented as a median (full range) due to unmeet normal distribution condition (alpha=0.05). Data were analyzed using Kruskal-Wallis test. B5 - Kruskal-Wallis test – 0.0265 / Kruskal-Wallis statistic 7.245; Mann-Whitney test: *CON vs 200 IS P = 0.0116; *100 IS vs 200 IS P = 0.0317. All Controls Kruskal-Wallis test – <0.0001 / Kruskal-Wallis statistic 20.91. All 100 IS Kruskal-Wallis test – 0.0087 / Kruskal-Wallis statistic 9.491. All 200 IS Kruskal-Wallis test – <0.0001 / Kruskal-Wallis statistic 22.09. (**c**) Data are presented as a median (full range) due to unmeet normal distribution condition (alpha = 0.05). Data were analyzed using Kruskal-Wallis test. B3 - Kruskal-Wallis test – 0.0352 / Kruskal-Wallis statistic 6.655; Mann-Whitney test: *CON vs 100 IS P = 0.032; All Controls Kruskal-Wallis test – 0.0168 / Kruskal-Wallis statistic 8.183. B1 – cerebellum; B2 – brainstem; B3 – cerebral cortex; B4 – hypothalamus; B5 – striatum with hippocampus; DA – dopamine; DOPAC – 3,4-dihydroxyphenylacetic acid; DOPAC/DA ratio – DA turnover; CON – control group; 100 IS – group receiving IS in the dose of 100 mg/kg b.w./day; 200 IS – group receiving IS in the dose of 200 mg/kg b.w./day; IS – indoxyl sulfate.

Chronically administered IS reduced DA concentration only in the brainstem – B2 part (P = 0.0244, 100 IS and P = 0.0304, 200 IS) (Fig. 3a). In turn, DOPAC concentration was decreased only in the B5 part that includes striatum with the hippocampus (P = 0.0116, 200 IS) (Fig. 3b). DOPAC/DA ratio reflected DA turnover showed no changes except a decrease in the cerebral cortex (B3 part) in 100 IS group (P = 0.032) (Fig. 3c). The level of DA in the B1 part and level of DOPAC in the B1 and B2 parts were below the detection limit.

#### Regional 5HT, 5HIAA levels, and 5HT turnover

**Figure 4:**
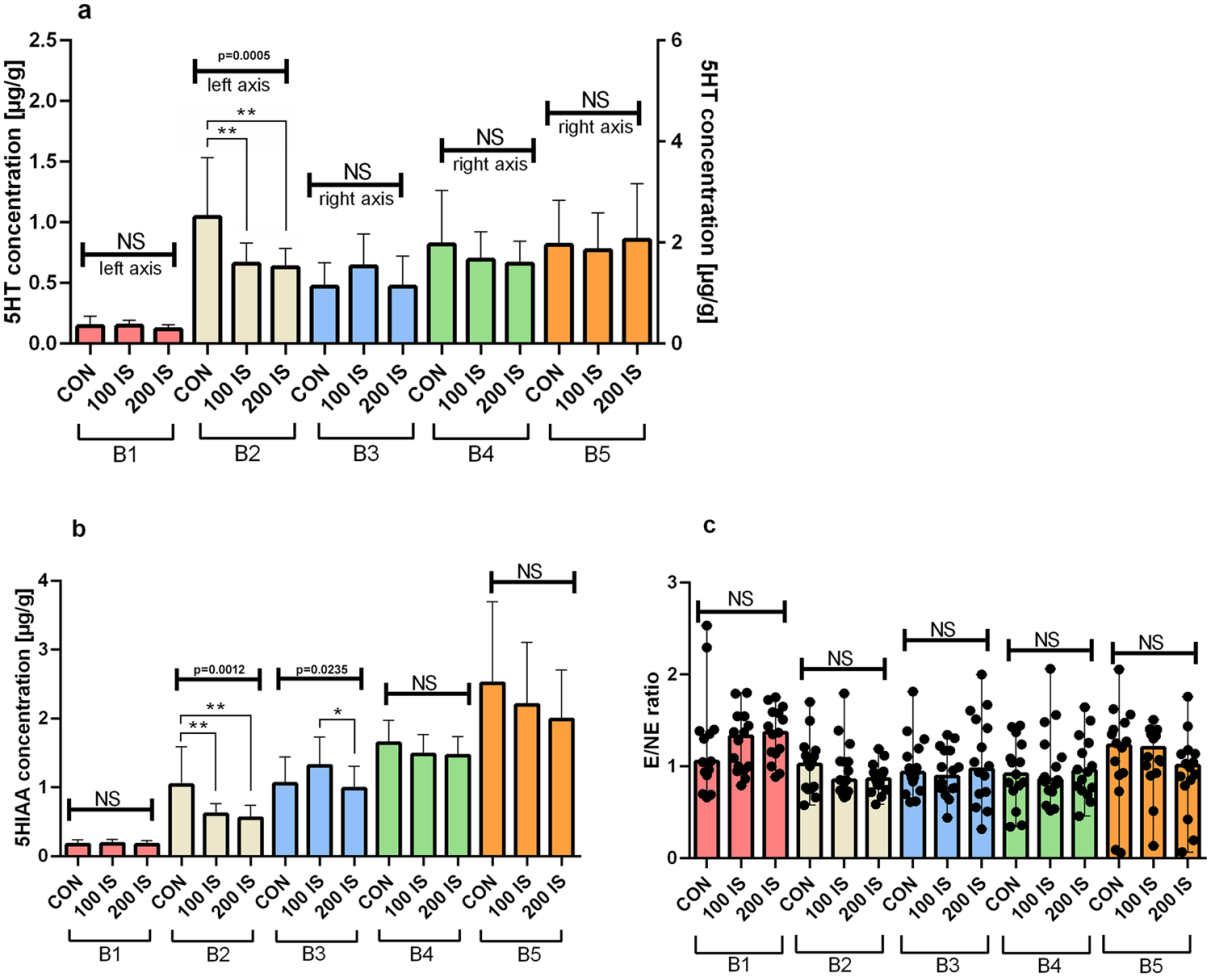
The effect of IS on regional 5HT (**a**), 5HIAA levels (**b**), and 5HT turnover (**c**); n = 45. (**a**) Data are presented as the mean ± SD due to passed normality test (alpha=0.05). Ordinary ANOVA test has been used to compare the data. B2 – Anova – 0.0005 / F (DFn, DFd) = F (2, 42) = 9.305; Student t-test: **CON vs 100 IS P = 0.0057; **CON vs 200 IS P = 0.0035. All Controls Anova – <0.0001 / F (DFn, DFd) = F (4, 70) = 20.01. All 100 IS Anova – <0.0001 / F (DFn, DFd) = F (4, 70) = 31.13. All 200 IS Anova – <0.0001 / F (DFn, DFd) = F (4, 70) = 24.43. (**b**) Data are presented as the mean ± SD due to passed normality test (alpha=0.05). Ordinary ANOVA test has been used to compare the data. B2 – Anova – 0.0012 / F (DFn, DFd) = F (2, 42) = 7.965; Student t-test: **CON vs 100 IS P = 0.0063; **CON vs 200 IS P = 0.0027. B3 - Anova – 0.0235 / F (DFn, DFd) = F (2, 42) = 4.105; Student t-test: *100 IS vs 200 IS P = 0.0148. All Controls Anova – <0.0001 / F (DFn, DFd) = F (4, 70) = 27.59. All 100 IS Anova – <0.0001 / F (DFn, DFd) = F (4, 70) = 40.52. All 200 IS Anova – <0.0001 / F (DFn, DFd) = F (4, 70) = 52.14. (**c**) Data are presented as a median (full range) due to unmeet normal distribution condition (alpha=0.05). Data were analyzed using Kruskal-Wallis test. All 100 IS Kruskal-Wallis test– 0.0229 / Kruskal-Wallis statistic 11.35. All 200 IS Kruskal-Wallis test – 0.0052 / Kruskal-Wallis statistic 14.78. B1 – cerebellum; B2 – brainstem; B3 – cerebral cortex; B4 – hypothalamus; B5 – striatum with hippocampus; 5HT – serotonin; 5HIAA – 5-hydroxyindolacetic acid; 5HIAA/5HT ratio – 5HT turnover; CON – control group; 100 IS – group receiving IS in the dose of 100 mg/kg b.w./day; 200 IS – group receiving IS in the dose of 200 mg/kg b.w./day; IS – indoxyl sulfate.

**Figure 5:**
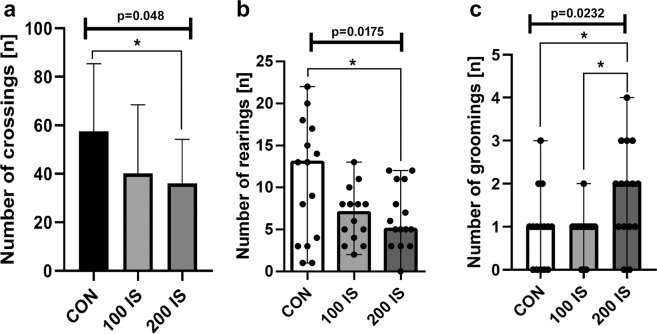
The effect of IS on the number of crossings (**a**), rearings (**b**), and groomings (**c**) in the open field test; n = 44. (**a**) Data are presented as the mean ± SD due to passed normality test (alpha=0.05). Ordinary ANOVA test has been used to compare the data. Anova – 0.048 / F (DFn, DFd) = F (2, 42) = 3.225; Student t-test: *CON vs 200 IS P = 0.0181. (**b**) Data are presented as a median (full range) due to unmeet normal distribution condition (alpha = 0.05). Data were analyzed using Kruskal-Wallis test. Kruskal-Wallis test – 0.0175 / Kruskal-Wallis statistic 8.092; Mann-Whitney test: *CON vs 200 IS P = 0.048. (**c**) Data are presented as a median (full range) due to unmeet normal distribution condition (alpha = 0.05). Data were analyzed using Kruskal-Wallis test. Kruskal-Wallis test – 0.0232 / Kruskal-Wallis statistic 7.529; Mann-Whitney test: *CON vs 200 IS P = 0.0371; *100 IS vs 200 IS P = 0.0103. CON – control group; 100 IS – group receiving IS in the dose of 100 mg/kg b.w./day; 200 IS – group receiving IS in the dose of 200 mg/kg b.w./day.

As shown in Figs. 4a,b, 5HT and 5HIAA concentrations were decreased after IS treatment only in the brainstem - B2 part (5HT – P = 0.0057, 100 IS and P = 0.0035, 200 IS; 5HIAA – P = 0.0063, 100 IS and P = 0.0027, 200 IS.). The chronic administration of IS did not alter 5HT turnover reflected by the 5HIAA/5HT ratio (Fig. 4c).

### Effects of the chronic exposure to IS on behavior

#### Open Field with Illuminated Center test

In the open field test, the number of crossings and rearings in the 200 IS group were lower than control (P = 0.0181 and P = 0.048, respectively) (Fig. 5a,b). In turn, the number of groomings after exposure to IS in the highest dose was increased compared to the control (P = 0.0371) and 100 IS group (P = 0.0103) (Fig. 5c). However, no significant effect was obtained for freezing, defecation, and micturition.

#### Elevated Plus Maze test

**Figure 6:**
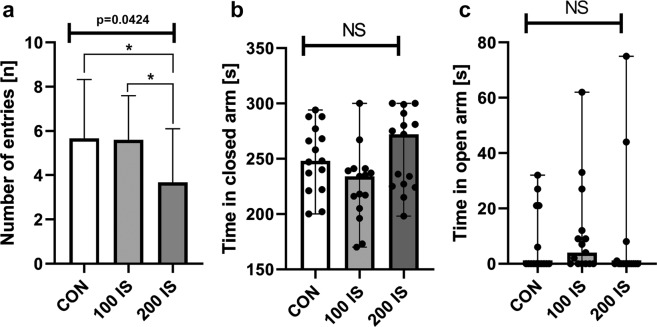
The effect of IS on the number of entries (**a**), time in closed (**b**) and open (**c**) arm in the elevated plus maze test; n = 45. (**a**) Data are shown as the mean ± SD. Anova – 0.0424 / F (DFn, DFd) = F (2, 42) = 3.412; Student t-test: *CON vs 200 IS P = 0.0408; *100 IS vs 200 IS P = 0.0245. (**b**) Data are presented as a median (full range) due to unmeet normal distribution condition (alpha = 0.05). Data were analyzed using Kruskal-Wallis test. (**c**) Data are presented as a median (full range) due to unmeet normal distribution condition (alpha = 0.05). Data were analyzed using Kruskal-Wallis test. CON – control group; 100 IS – group receiving IS in the dose of 100 mg/kg b.w./day; 200 IS – group receiving IS in the dose of 200 mg/kg b.w./day; IS – indoxyl sulfate.

Locomotor activity reflected by the number of entries was significantly reduced in the 200 IS group (Fig. 6a) compared to the control and 100 IS group (P = 0.0408 and P = 0.0245, respectively). Moreover, we did not notice any differences in time spent in closed or open arms (Fig. 6b,c).

#### Chimney test

**Figure 7:**
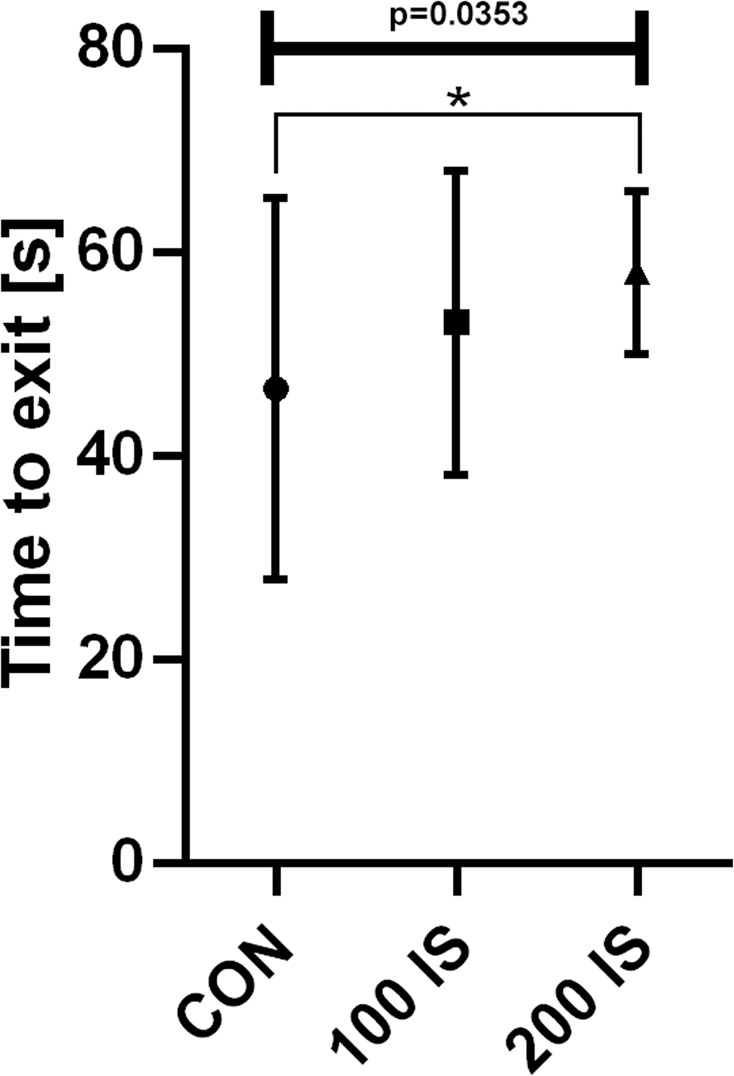
The effect of IS on time to exit in chimney test; n = 45. Data are presented as a median (full range) due to unmeet normal distribution condition (alpha = 0.05). Data were analyzed using Kruskal-Wallis test. Kruskal-Wallis test – 0.0353 / Kruskal-Wallis statistic 6.688; Student t-test: *CON vs 200 IS P = 0.0133.

Chronic administration of IS in the dose of 200 mg/kg of b.w./day resulted in a slight motor impairment, which was illustrated by increased time to exit (P = 0.0133) (Fig. 7).

#### T Maze test

**Figure 8:**
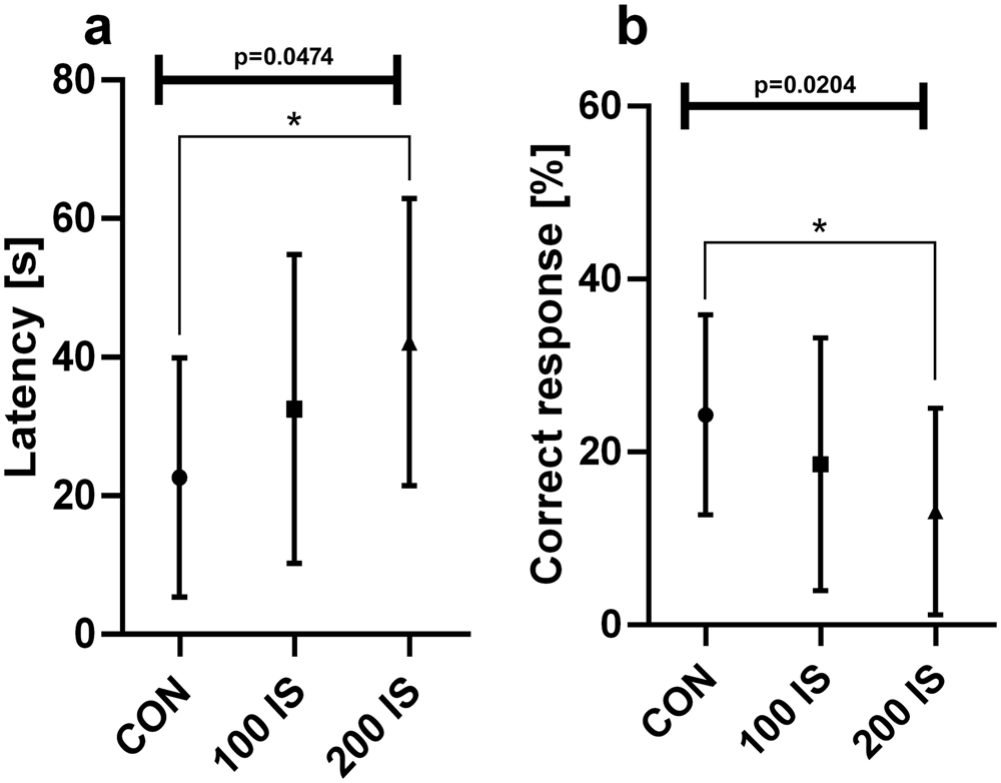
The effect of IS on the latency (**a**) and percentage of the correct response (**b**) in the T maze test; n = 45. Data are presented as a median (full range) due to unmeet normal distribution condition (alpha = 0.05). Data were analyzed using Kruskal-Wallis test. (**a**) Kruskal-Wallis test – 0.0474 / Kruskal-Wallis statistic 6.124; Student t-test: *CON vs 200 IS P = 0.0182. (**b**) Kruskal-Wallis test – 0.0204 / Kruskal-Wallis statistic 7.789; Student t-test: *CON vs 200 IS P = 0.0116. CON – control group; 100 IS – group receiving IS in the dose of 100 mg/kg b.w./day; 200 IS – group receiving IS in the dose of 200 mg/kg b.w./day; IS – indoxyl sulfate.

As shown in Fig. 8, rats from 200 IS group were characterized by longer latency to complete a session (Fig. 8a) and a decrease in the percentage of correct response (Fig. 8b) compared to the control group (P = 0.0182 and P = 0.0116, respectively).

#### Splash test

**Figure 9:**
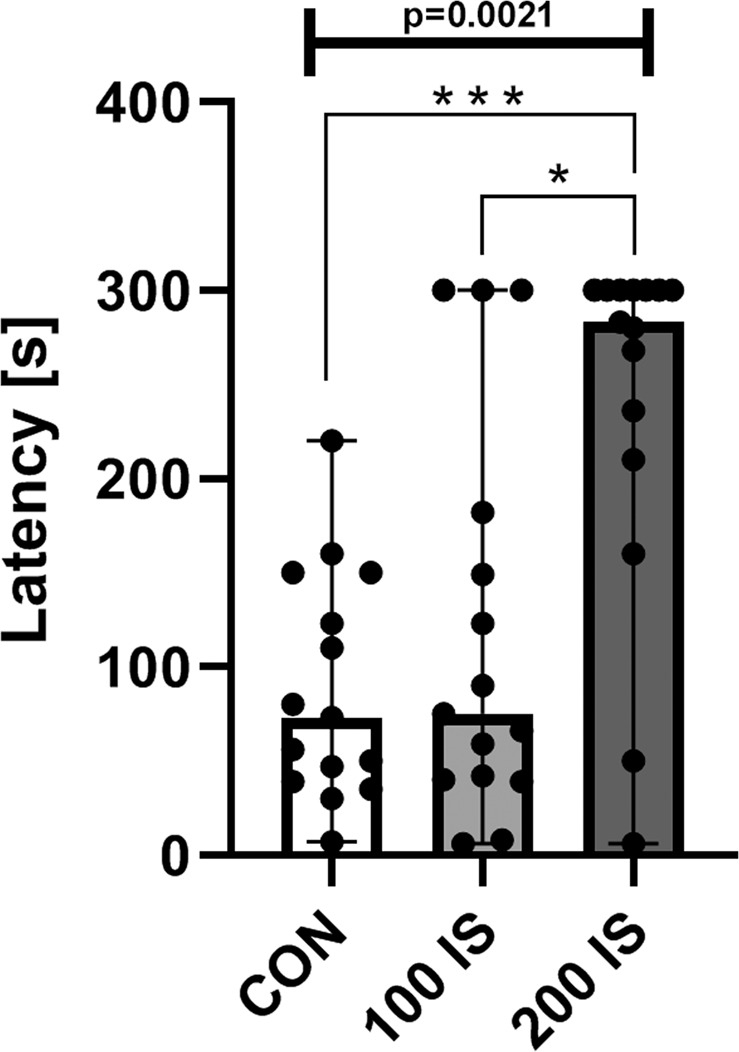
The effect of IS on the latency in the splash test; n = 45. Data are presented as a median (full range) due to unmeet normal distribution condition (alpha = 0.05). Data were analyzed using Kruskal-Wallis test. Kruskal-Wallis test – 0.0021 / Kruskal-Wallis statistic 12.31; Mann-Whitney test: ***CON vs 200 IS P = 0.0002; *100 IS vs 200 IS P = 0.0172. CON – control group; 100 IS – group receiving IS in the dose of 100 mg/kg b.w./day; 200 IS – group receiving IS in the dose of 200 mg/kg b.w./day; IS – indoxyl sulfate.

The results of the splash test revealed that rats exposed to IS in the dose of 200 mg/kg of b.w./day exhibited significantly increased idle time between the first spray and initiation of grooming (latency) compared to control (P = 0.0002) and 100 IS group (P = 0.0172) (Fig. 9).

### Relationships

#### Relationships between cerebral IS concentrations and monoamines levels

**Figure 10:**
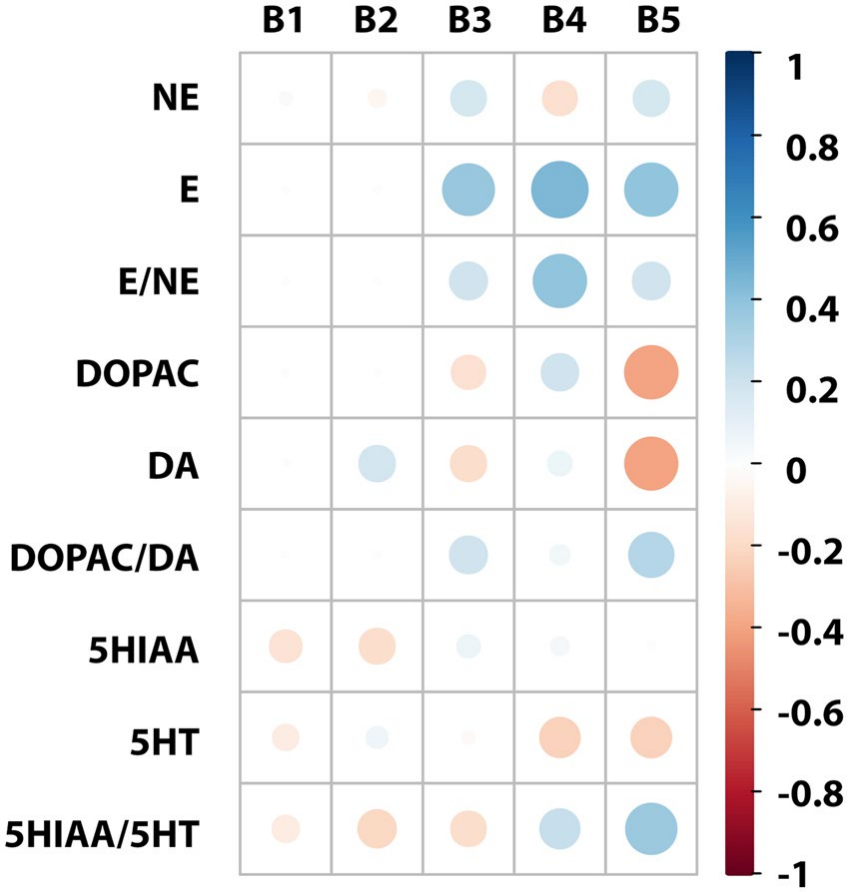
Correlation matrix between cerebral IS concentrations and monoamines levels. The size of the circle and intensity of color represent the strength of the correlation (darker and larger circles demonstrate the strong correlation). Blue colors – positive correlations; red colors – negative correlations. B1 – cerebellum; B2 – brainstem; B3 – cerebral cortex; B4 – hypothalamus; B5 – striatum with hippocampus; NE – norepinephrine; E – epinephrine; E/NE – NE turnover; DA – dopamine; DOPAC – 3,4-dihydroxyphenylacetic acid; DOPAC/DA – DA turnover; 5HT – serotonin; 5HIAA – 5-hydroxyindolacetic acid; 5HIAA/5HT – 5HT turnover.

Brain concentration of IS correlated positively with levels of E in the cortex (B3 part), hypothalamus (B4 part), and striatum with the hippocampus (B5 part). Positive correlations were also observed between IS level and E/NE ratio in hypothalamus, as well as between IS level and 5HIAA/5HT ratio in the striatum with hippocampus. Both DOPAC and DA levels were inversely associated with IS concentrations in the striatum with hippocampus (Fig. 10).

#### Relationships between cerebral IS concentrations and behavioral tests

**Figure 11:**
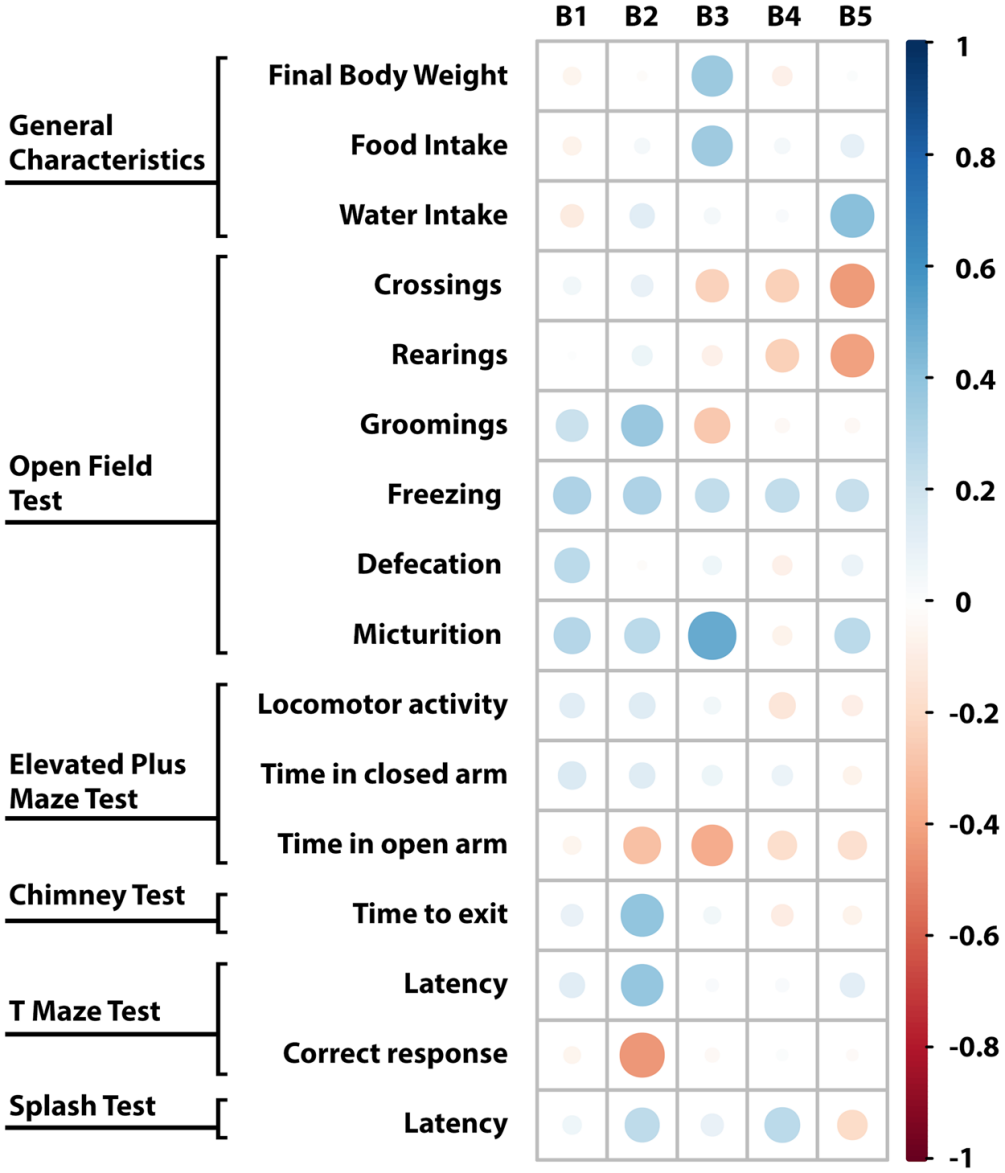
Correlation matrix between cerebral IS concentrations and behavioral tests. The size of the circle and intensity of color represent the strength of the correlation (darker and larger circles demonstrate the strong correlation). Blue colors - positive correlations; red colors – negative correlations. B1 – cerebellum; B2 – brainstem; B3 – cerebral cortex; B4 – hypothalamus; B5 – striatum with hippocampus.

The cerebellum and hypothalamus IS levels did not correlate with any parameters of behavioral tests. The only tendency to a positive correlation was observed between the cerebellum IS level and freezing (open field test) (r = 0.3062, p = 0.09). In turn, brainstem IS concentration correlated positively with groomings (open field test), time to exit (chimney test), and latency (T maze test), as well as inversely with the correct response (T maze test). In this case, we also noticed the tendency to a positive correlation with freezing (open field test) (r = 0.3111, p = 0.09). Concerning to the cerebral cortex IS level, we observed a positive association with final body weight, food intake, micturition (open field test), and time in open arm (elevated plus maze test). Moreover, we found a positive correlation between the concentration of IS in the striatum with hippocampus and water intake, and a negative correlation between IS level in this part of brain and crossings, and rearings (open field test) (Fig. 11).

#### Relationships between cerebral IS levels and plasma IS concentrations

As shown in Table 2, plasma IS concentrations^12^ correlated positively with cerebral IS levels. The strongest correlation was found between plasma IS concentration and IS level in the striatum with hippocampus (B5 part).

**Table 2:** Relationships between cerebral IS levels and plasma IS concentrations.

	Correlation coefficient, R	p-value
B1	0.4145	0.0052
B2	0.4798	0.0010
B3	0.4169	0.0054
B4	0.4211	0.0044
B5	0.5896	<0.0001

B1 – cerebellum; B2 – brainstem; B3 – cerebral cortex; B4 – hypothalamus; B5 – striatum with hippocampus; IS – indoxyl sulfate. Bold values mean that the correlation was statistically significant.

### Lipid peroxidation

**Figure 12:**
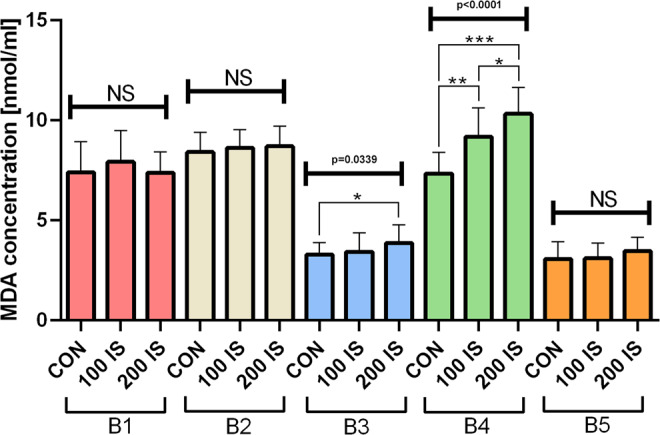
The effect of IS on MDA concentrations; n = 45. Data are presented as the mean ± SD due to passed normality test (alpha=0.05). Ordinary ANOVA test has been used to compare the data. B3 - Anova – 0.0339 / F (DFn, DFd) = F (2, 42) = 3.674; Student t-test: *CON vs 200 IS P = 0.0431. B4 - Anova – <0.0001 / F (DFn, DFd) = F (2, 42) = 24.53; Student t-test: **CON vs 100 IS P = 0.004; ***CON vs 200 IS P < 0.0001; *100 IS vs 200 IS P = 0.0276. All Controls Anova – <0.0001 / F (DFn, DFd) = F (4, 70) = 82.41. All 100 IS Anova – <0.0001 / F (DFn, DFd) = F (4, 70) = 110. All 200 IS Anova – <0.0001 / F (DFn, DFd) = F (4, 70) = 137.9. B1 – cerebellum; B2 – brainstem; B3 – cerebral cortex; B4 – hypothalamus; B5 – striatum with hippocampus; CON – control group; 100 IS – group receiving IS in the dose of 100 mg/kg b.w./day; 200 IS – group receiving IS in the dose of 200 mg/kg b.w./day; IS – indoxyl sulfate; MDA – malondialdehyde.

The concentrations of lipid peroxidation products reflected by MDA concentrations were increased only in the cortex – B3 part in 200 IS group (P = 0.0431) and hypothalamus – B4 part in both IS treated groups (P = 0.004, 100 IS and P < 0.0001, 200 IS) (Fig. 12).

## Discussion

The present study has confirmed the ability of IS to accumulate in the brain tissue and has revealed IS effect on behavioral profile and cerebral monoamines, providing evidence of IS neurotoxic role. Moreover, for the first time, the IS levels were evaluated separately in the cerebellum, brainstem, cortex, hypothalamus, and striatum with the hippocampus that allowed to observe the highest IS accumulation in the brainstem. Unexpectedly, we did not notice increased IS accumulation in the hypothalamus. Additionally, our results show that chronic exposure to IS leads to reduced locomotor activity and spatial memory, as well as increased stress sensitivity, and apathetic behavior. Besides the behavioral disturbances, we observed slight alterations in the cerebral monoamines that were reflected by reduced levels of NE, DA or 5HT, mainly in the brainstem.

CKD is connected with a higher risk of neurological complications with various etiologies^17^. In addition to cerebrovascular disease, secondary hyperparathyroidism, and anemia, CKD lead to the accumulation of uremic toxins, which can result in toxic-metabolic encephalopathy^4,5^. IS is one of the potent uremic toxins that can contribute to the disruption of CNS homeostasis. Its ability to accumulate in the brain tissue was shown by Zgoda-Pols *et al*.^16^ after chemically-induced acute kidney injury in mice and by Iwata *et al*.^14^ after cisplatin-induced acute renal failure. In the present study, we have also confirmed it. However, for the first time, we show IS concentrations in particular parts of the brain like cerebellum (B1), brainstem (B2), cortex (B3), hypothalamus (B4), and striatum with the hippocampus (B5). Furthermore, as we showed in our previous work, excretory kidney function remains vital and efficient in our model^12^. Therefore, the model enabled to observe primarily influence of IS without greater interferences of other uremic toxins, which can change the integration of the blood-brain barrier, and therefore facilitate IS brain accumulation. Two IS doses (100 and 200 mg/kg of b.w./day) administered chronically led to increased plasma IS concentrations that reflect IS concentrations of CKD patients^18^. What is important, plasma IS concentrations correlated positively with cerebral IS levels.

Altered mental status, which occurs in CKD, can include confusion, disorientation, amnesia, restlessness, behavior changes, and other cognitive dysfunctions, as well as disruptions in psychomotor skills^1,5^. Available animal behavioral studies show similar disturbances involving depression-like and anxiolytic behavior, decreased locomotor and emotional activity, as well as reduced exploratory drive after surgical or chemical induction of chronic renal insufficiency^19–21^. Similar results were obtained after chronic exposure to IS in the present work, which supports IS behavioral neurotoxicity. First of all, we found reduced locomotor activity reflected by decreased number of crossings in the open field test and reduced total number of entries in the elevated plus maze test, which can also be connected with a slight motor impairment (observed in the chimney test), as well as apathetic behavior (confirmed by the splash test). Moreover, the decreased exploratory activity can also be associated with the mentioned locomotor activity. However, the common measure of exploratory behavior is rearings^22^. Importantly, our observation of a reduction in this parameter is in line with the results of behavioral study using CKD animal model^21^.

Rearing can be linked not only to vigilance and exploratory behavior, but also anxiety. It is said that stress reduces the number of rearings in the open field test, thus obtained data can indicate the increased anxiety after chronic exposure to IS in our work. Another measure of anxiogenic response is grooming that is considered as a mechanism to alleviate anxiety^23,24^. Chronic administration of IS increased in both several spontaneous (stress-evoked) grooming in the open field test and latency to artificial groom in the splash test. Since excessive grooming is specific to stressed rats, we can also say that rats chronically exposed to IS were characterized by enhanced emotional stress reactions and increased stress sensitivity. However, even though open field test indicated meaningful changes that can reflecting increased anxiety, these results are not entirely supported by our findings from the elevated plus maze test. Although we found the negative correlation between time spent in the open arm and IS concentration in the cortex, the exposure to IS did not significantly affect the time spent in the open or closed arm. Therefore, we cannot clearly state that IS potentiates anxiety. Besides ambulation, percent of time spent in central and peripheral zones should be measured in the open field test in the future studies.

Apart from decreased locomotor activity, slight impairment of motor coordination, and increased stress sensitivity, we also observed a decrease in the percent of correct responses and longer latency to complete the session in the T maze test, which suggests the impairment of spatial memory in rats exposed to IS. Moreover, the concentration of IS in the brainstem correlated inversely with correct responses and positively with the latency to complete the session. Cognitive impairment including memory, executive functioning, orientation, language, and visual-spatial learning is common in patients with CKD^5,17^. Therefore, further more specific behavioral tests are necessary to assess IS effect on other cognitive function.

Behavioral changes observed during CKD may be the result of neurotransmitters derangements. Hitherto, described changes in cerebral neurotransmitters in animal models with kidney failure are mainly associated with depletion of brain catecholamines like NE, E, and DA^3,20,25^. Data from our study coincide to some extent with them. We have noticed a decrease in NA concentrations in the cerebellum and brainstem. Interestingly, we did not observe any changes in E concentrations. However, we were not able to evaluate E concentrations in the cerebellum and brainstem due to the limit of detection. Despite the lack of changes, E concentrations correlated positively with IS levels in the cortex, hypothalamus, and striatum with the hippocampus. Beside this, we also noticed a positive correlation between NE turnover and IS concentrations in the hypothalamus.

Additionally, we observed the reduction of DA concentration in the brainstem. Adachi *et al*.^20^ showed decreased DA turnover in the striatum, mesencephalon, and hypothalamus, and concluded that uremia is connected with the suppression of the central dopaminergic metabolism that results in disturbed motor activity. Despite decreased locomotor activity after chronic administration of IS, our results did not demonstrate any differences in DA turnover. Obtained data concerning catecholamines did not allow for a full explanation of behavioral disturbances after chronic exposure to IS based on catecholamines changes. However, we cannot completely exclude their impact on behavioral alterations. Undoubtedly, additional studies are required for further understanding of the importance of these changes.

Another monoamine brain level that was evaluated during our research was 5HT level. Available data concerning its cerebral changes are contradictory. On the one hand, Siassi *et al*.^26^ observed an increase in the brain 5HT concentration and 5HT turnover, but on the other hand, Adachi *et al*.^20^ did not show any changes in them during CKD. The differences may be connected with the usage of various animal models. However, chronic exposure to IS in our study did not affect 5HT concentrations in all parts of the brain, except the brainstem, where 5HT concentration was reduced. Moreover, chronic administration of IS did not alter 5HT turnover. Generally, high brain serotonin levels are considered as a cause of anorexia in CKD. The work of Topczewska-Bruns *et al*.^21^ showed a reduction in food intake and body weight of rats with chronic renal insufficiency. In this context, our result concerning cerebral 5HT concentration and turnover is consistent with the lack of differences in food intake and final body weight between IS groups and control.

IS has been reported to induce oxidative stress and inflammatory mediators in glial cells^9^. Therefore, IS-induced oxidative stress and lipid peroxidation may contribute to the neuronal degeneration and neurological complications observed in CKD patients. In our study we found increased lipid peroxidation only in the cortex and hypothalamus of rats exposed to IS. However, due to limited amount of brain tissue we were able to assess MDA concentrations only. Our research has a primarily descriptive character – taking into account recent advances in the field, presented paper confirms preliminary clinical reports and to the best of our knowledge, is the first study broadly investigating links between IS and neurobehavioral environment. Further research is needed to explain IS influence on CNS disorders via induction of inflammation and oxidative stress.

In conclusion, this study demonstrates that chronic exposure to IS leads to behavioral alterations involving apathetic behavior, increased stress sensitivity, and reduced locomotor and exploratory activity. In addition to these, IS can contribute to the impairment of spatial memory and motor coordination. Despite observed disturbances cannot be explained completely by changes in cerebral monoamines concentrations and turnovers, our findings confirmed IS ability to accumulate in the brain tissue, and moreover, indicate that it can be one of the crucial uremic factors responsible for altered mental status in the course of CKD.

## Methods

### Animals

Albino Wistar rats (Wistar:cmd; outbred Cmdb:WI) of male sex (180–200 g) were exposed to 7 days acclimatization to the conditions of animal house at DLAR Animal Facility at Medical University of Bialystok, Poland. Well ventilation, 25 °C temperature, humidity (55 ± 5%) and 12-hour light and dark cycles were maintained carefully in animal house. Single caged rats were housed per individually ventilated cage (IVC). Standard pellet diet Ssniff R-Z V1324 (Ssniff Spezialdiäten GmbH, Germany) and sufficient water were provided to rats. All researchers obtained ethical clearance for conducting experiments on animals from Polish Laboratory Animal Science Association (PolLASA), and the research workflow was approved by the Institutional Local Ethics Committee of the University of Warmia and Mazury in Olsztyn (Agreements No. 124/2015, No. 31/2017/WNP), confirming that all experiments were performed in accordance with relevant guidelines and regulations. 3Rs principles were implemented within all the procedures during the study accordingly to Graham M. L. and Prescott M. J.^27^. The animals’ health status was monitored throughout the experiments by a health surveillance program according to Federation of European Laboratory Animal Science Associations (FELASA) guidelines. The rats were free of all viral, bacterial, and parasitic pathogens listed in the FELASA recommendations. The study was conducted by ARRIVE guidelines^28^ and directive 2010/63/EU of the European Parliament and of the Council on the protection of animals used for scientific purposes. Euthanization of all animals at the end of the experiment was performed using overdose of pentobarbital and pentobarbital natrium cocktail (Morbital, Biowet) and followed decapitation.

### Chemicals

All the chemicals and reagents used were commercially available and were of the highest grade purity or analytical purity. Indoxyl sulfate potassium salt, the monoamines (NE, E, DA, 5HT) and their metabolites (DOPAC, 5HIAA), sucrose, sodium acetate, EDTA-2NA were purchased from Sigma-Aldrich, USA. Acetic acid, acetonitrile, citric acid, HClO_4_, 1-octanesulfonic acid sodium were purchased from Merck, Germany.

### IS treatment and experimental design

The rats were randomly divided into three groups. Randomization was performed accordingly to Kim J. and Shin W.^29^. using Block Randomization approach after randomly assigned numbers to the rats undergoing experiment by another *blinded* person. After the allocation of animals in one of the three groups, we did not observe any differences in basic parameters in the course of quarantine period. IS doses were fixed by conducting preliminary dose dependent test as well as were based on our previous experimental work^11^. The doses of 100 and 200 mg/kg of IS were selected in the current investigation. IS in the doses of 100 mg/kg of body weight (b.w.)/day and 200 mg/kg of b.w./day were administered in the drinking water for 4 weeks to experimental groups named 100 IS and 200 IS, respectively. In turn, the control group received tap water without IS. The behavioral profile of animals in the chimney test, splash test, open field test, T maze test and elevated plus maze test was assessed between day 24 and day 27. All experiments were conducted between 9.00 and 17.00 hours by the same experimenter. After 4 weeks, rats were anesthetized with pentobarbital (40 mg/kg, ip) and decapitated. The brains were removed rapidly and dissected on ice into five parts: B1 (cerebellum), B2 (brainstem), B3 (cerebral cortex), B4 (hypothalamus) and B5 (striatum with hippocampus), which were immediately snap-frozen using liquid nitrogen and stored at −80 °C until high-performance liquid chromatography (HPLC) assays were performed.

### HPLC analyses of postmortem tissue

All the post-mortem assays using HPLC were conducted in triplicates, then the mean was withdrawn and used as a representing value. The order of analyzed samples was random. All the HPLC measurements were preceded and completed by quality control (QC) samples measurement. Additional QC procedures were performed in the middle of measurements.

#### Determination of IS in the brain regions

Brain levels of IS were assessed using HPLC with fluorescence detection (Agilent Technologies 1260 Infinity series) according to our modification of the methods previously described by Al Za’abi *et al*.^30^. The brain tissues were weighed and homogenized in acetonitrile containing methyl paraben (0.1 mg/mL) as an internal standard in a ratio of 1:5. The homogenates were placed at 4 °C for 60 min, and then the samples were centrifuged at 15,260 × g for 30 min at 4 °C (Eppendorf Centrifuge 5430 R). After centrifugation, the supernatant was filtered (0.45-mm Millipore filter) and injected (10 μL) into the HPLC system. The chromatographic separations were performed on the Aeris Peptide XB-C18 column (4.6 mm × 250 mm, 3.6 µm particle size, Phenomenex) at 24 °C. The mobile phase was composed of sodium acetate buffer (pH 4.5) and acetonitrile (10:90, v/v); the flow rate was 0.6 mL/min. The excitation and emission wavelengths were 280 nm and 375 nm, respectively.

#### Determination of monoamines in the brain regions

The monoamines (NE, E, DA, 5HT) and their metabolites (DOPAC, 5HIAA) were determined by HPLC method with electrochemical detection (Agilent Technologies 1260 Infinity series) according to the previously reported method of Zhang *et al*.^31^. The brain tissues were weighed and homogenized in a five-fold volume of 0.4 M HClO_4_ solution. The homogenates were placed at 4 °C for 60 min and then centrifuged at 15,260 × g for 30 min at 4 °C (Eppendorf Centrifuge 5430 R). The supernatant was filtered using a 0.45 µm Millipore filter. One µL of the solution was injected into the HPLC system. The separations were performed on the ODS2 column (2.1 mm × 150 mm, 3 µm particle size, Waters Spherisorb). The mobile phase was composed of a buffer solution (25 mM citric acid, 25 mM sodium acetate, 1.0 mM 1-octanesulfonic acid sodium, and 0.01 mM EDTA-2Na) adjusting pH with acetic acid to 3.35 and 10% acetonitrile, and was pumped at a flow rate of 0.1 mL/min. The potential of the working electrode was 0.7 V.

### Behavioral tests

#### Open field with illuminated center test

The open field test with the illuminated center is used to assess the level of exploration drive and anxiety level. The test creates conditions for the novelty of the environment, and is used to evaluate the level of stress sensitivity, especially for neurogenic stimuli, and to trigger an emotional stress reaction. The open field test determines not only the locomotor activity and the degree of exploration in the new environment, but also the level of anxiety towards the stress factor (of neurogenic nature), which is the open space.

The open field test was conducted in boxes made of a white laminated board whose dimensions are 100 × 100 × 60 cm, with the floor divided into 25 equally sized squares measuring 20 × 20 cm each. A 200 watt light bulb was placed above each box. The research was carried out at the same time between 8:00 and 12:00. During the experiment, the animals’ behavior was characterized by the total number of crossings (the general locomotion measured by the number of crossed squares), rearing (the number of episodes of standing on the hind paws and body verticalization), grooming (the number of fur cleaning episodes and cleaning muzzles), freezing (the duration in minutes of motionlessness), defecation (the number of defecation episodes), micturition (the number of episodes of micturition). Rodents were placed in the same position in the middle of the box. The behavior of the rats was monitored for 10 minutes. Two observers, who were in the same places, observed behavior of animals on the ethograms.

#### Elevated plus maze test

The elevated plus maze test measures anxiety-like behavior. The test is based on the habitual preference for a dark and natural aversion of rats for open and elevated areas, as well as on their natural spontaneous exploratory behavior in novel environments.

The apparatus consists of open arms and closed arms, crossed in the middle perpendicularly to each other, and a center area. Rats were given access to all of the arms and are allowed to move freely between them. According to Pellow^32^, each rat was placed in a 60 × 60 × 35 cm wooden box for 5 minutes before the actual test. After the adaptation period, each rat was placed individually at the crossroads of the open and closed arms of the cross maze (mounted on a stand, 50 cm from the floor) with the head facing the closed arm. Within 5 minutes of the test number of entries into open arms, time spent in open arms, the number of entries into closed arms, and time spent in closed arms were measured by two observers sitting 1 m from the open arms of the labyrinth. The tests were carried out each time for the control and test group. Assessed parameters include the total number of entries which reflect locomotor activity and the time spent in the open and closed arm as the indicators of open space-induced anxiety in mice.

#### Chimney test

The motor coordination was assessed in the chimney test according to the method described by Boissier *et al*.^33^. Each animal was individually placed head-down in a plastic tube (internal diameter 57 mm and length 452 mm). The test consisted in determining the time that the rat needed to climb backwards up the tube. Motor impairment was determined by the inability to perform the task in less than 60 seconds.

#### T maze test

T maze test, which is based on the premise that animals have evolved an optimal strategy to explore their environment and obtain food with a minimum amount of effort, was used for the assessment of spatial memory.

Animals were started from the base of the T segment and allowed to choose one of the goal arms. The prize (food) was placed in one arm of the labyrinth. The rat approached and chose the left or right arm of the labyrinth. Each trial should have been completed in under 1 min. The day before the test, the animals underwent initial training. The percentage of correct response and latency to complete a session were assessed.

#### Splash test

The splash test was performed under red light (230 V, 15 W) and consisted of squirting a 10% sucrose solution on the dorsal coat of rats. Due to its viscosity, the sucrose solution dirties the fur that initiates artificial grooming behavior that can be deemed as an index of self-care and motivational behavior. Latency to groom was measured after the vaporization of the sucrose solution. An increase in latency to the groom is comparable to some symptoms of depression, such as apathetic behavior.

### Malondialdehyde (MDA) determination

The method of Buege and Aust^34^ was used for the determination of MDA levels in the brain that reflect lipid peroxidation. 10% homogenates of the brain tissue were prepared in ice-cold 0.9% NaCl using a homogenizer (Dounce homogenizer; Schütt Labortechnik GmbH, Göttingen, Germany). Next, the homogenates were centrifuged (Eppendorf Centrifuge 5430 R) at 6,000 × g for 20 min. The concentration of MDA was determined as thiobarbituric acid reactive substances (TBARS) and measured the level of product at 532 nm using a Hitachi U-3010 Spectrophotometer (Japan).

### Statistical approach and analysis

Numbers of rats to be used for each group in studies were estimated and based on our published and new preliminary data, and assuming a power of 0.9 to detect a difference of at least 20% with significance of 0.05. The calculation of statistical power was made using GrapdPad StatMate 2 Software (La Jolla, CA, USA). After statistical analysis all the results were subjected to checking the statistical power of performed analysis. Presented data had at least 80% power to detect a difference between means of high scientifically significance level (alpha) of 0.05 (two-tailed). All statistical analyses were performed using GraphPad Prism 8 (GraphPad Software, La Jolla, CA, USA). Checking the distribution before performing the competent statistical analysis has been performed using Shapiro-Wilk Normality test followed by Student’s t-test for two subsets of data or one-way ANOVA (when comparing more than three subtypes of variables) when data follow a normal distribution, and nonparametric Mann-Whitney U test or Kruskall-Wallis test (skewed distribution), and followed by Bonferroni post hoc analysis when appropriate. The normally distributed data were presented as mean ± SD, while the non-Gaussian data as median (full-range). The correlations between study variables in IS-treated groups were calculated by Spearman’s rank correlation (nonparametric set of the data) or Pearson correlation coefficient analyses when all subsets of data met normal distribution condition. P-value ˂0.05 was considered statistically significant. Graphic design presentation of results was prepared using GraphPad Prism 8 or R statistical software (version 3.5.0). The Fig. 1 was prepared using Adobe Photoshop CC.

## References

[1] Arnold R, Issar T, Krishnan AV, Pussell BA (2016). Neurological complications in chronic kidney disease. JRSM Cardiovasc. Dis..

[2] Baumgaertel, M. W., Kraemer, M. & Berlit, P. Neurologic complications of acute and chronic renal disease in Handbook of clinical neurology (eds. J., Biller & J. M., Ferro) 383-393 (Elsevier, 2014). 10.1016/B978-0-7020-4086-3.00024-2 24365307

[3] Nongnuch, A. Panorchan, K. & Davenport, A. Brain-kidney crosstalk. *Crit. Care* **225**; 10.1186/cc13907 (2014). 10.1186/cc13907 PMC4075125 25043644

[4] Baluarte JH (2017). Neurological complications of renal disease. Semin. Pediatr. Neurol..

[5] Watanabe K, Watanabe T, Nakayama M (2014). Cerebro-renal interactions: impact of uremic toxins on cognitive function. Neurotoxicology.

[6] Niwa, T. Indoxyl sulfate is a nephro-vascular toxin. *J. Ren. Nutr*. **S2–6**; 10.1053/j.jrn.2010.05.002 (2010). 10.1053/j.jrn.2010.05.002 20797565

[7] Vanholder, R., Pletinck, A., Schepers, E. & Glorieux, G. Biochemical and clinical impact of organic uremic retention solutes: a comprehensive update. *Toxins* **E33**; 10.3390/toxins10010033 (2018). 10.3390/toxins10010033 PMC5793120 29316724

[8] Ohtsuki S (2002). Role of blood-brain barrier organic anion transporter 3 (OAT3) in the efflux of indoxyl sulfate, a uremic toxin: its involvement in neurotransmitter metabolite clearance from the brain. J. Neurochem..

[9] Adesso S (2017). Indoxyl sulfate affects glial function increasing oxidative stress and neuroinflammation in chronic kidney disease: interaction between astrocytes and microglia. Front. Pharmacol..

[10] Watanabe I (2013). Activation of aryl hydrocarbon receptor mediates indoxyl sulfate-induced monocyte chemoattractant protein-1 expression in human umbilical vein endothelial cells. Circ. J..

[11] Karbowska, M. *et al*. The uremic toxin indoxyl sulfate accelerates thrombotic response after vascular injury in animal models. *Toxins* **E229**; 10.3390/toxins9070229 (2017). 10.3390/toxins9070229 PMC5535176 28753957

[12] Karbowska, M. *et al*. Indoxyl sulfate promotes arterial thrombosis in rat model via increased levels of complex TF/VII, PAI-1, platelet activation as well as decreased contents of SIRT1 and SIRT3. *Front. Physiol*. **1623**; 10.3389/fphys.2018.01623 (2018). 10.3389/fphys.2018.01623 PMC6279869 30546314

[13] Gondouin B (2013). Indolic uremic solutes increase tissue factor production in endothelial cells by the aryl hydrocarbon receptor pathway. Kidney Int..

[14] Iwata K (2007). Involvement of indoxyl sulfate in renal and central nervous system toxicities during cisplatin-induced acute renal failure. Pharm. Res..

[15] Wang G, Korfmacher WA (2009). Development of a biomarker assay for 3-indoxyl sulfate in mouse plasma and brain by liquid chromatography/tandem mass spectrometry. Rapid Commun. Mass Spectrom..

[16] Zgoda-Pols JR (2011). Metabolomics analysis reveals elevation of 3-indoxyl sulfate in plasma and brain during chemically-induced acute kidney injury in mice: investigation of nicotinic acid receptor agonists. Toxicol. Appl. Pharmacol..

[17] Yeh YC (2016). Indoxyl sulfate, not p-cresyl sulfate, is associated with cognitive impairment in early-stage chronic kidney disease. Neurotoxicology.

[18] Kaminski, T. W., Pawlak, K., Karbowska, M., Mysliwiec, M. & Pawlak, D. Indoxyl sulfate – the uremic toxin linking hemostatic system disturbances with the prevalence of cardiovascular disease in patients with chronic kidney disease. *BMC Nephrol*. **35**; 10.1186/s12882-017-0457-1 (2017). 10.1186/s12882-017-0457-1 PMC5267373 28122514

[19] Al Banchaabouchi M, D’Hooge R, Marescau B, De Deyn PP (1999). Behavioural deficits during the acute phase of mild renal failure in mice. Metab. Brain Dis..

[20] Adachi N (2001). Uraemia suppresses central dopaminergic metabolism and impairs motor activity in rats. Intensive Care Med..

[21] Topczewska-Bruns J, Tankiewicz A, Pawlak D, Buczko W (2001). Behavioral changes in the course of chronic renal insufficiency in rats. Pol. J. Pharmacol..

[22] Alves R, Barbosa de Carvalho JG, Venditti MAC (2012). High- and low-rearing rats differ in the brain excitability controlled by the allosteric benzodiazepine site in the GABA_A_ receptor. J. Behav. Brain Sci..

[23] Hu, C. *et al*. Re-evaluation of the interrelationships among the behavioral tests in rats exposed to chronic unpredictable mild stress. *PLoS One***e0185129**; 10.1371/journal.pone.0185129 (2017). 10.1371/journal.pone.0185129 PMC5607203 28931086

[24] Smolinsky, A. N., Bergner, C. L., LaPorte, J. L. & Kalueff, A. V. Analysis of grooming behavior and its utility in studying animal stress, anxiety, and depression in Mood and anxiety related phenotypes in mice. Neuromethods. (ed. T., Gould) 21–36 (Humana Press 2009).

[25] Ali F, Tayeb O, Attallah A (1985). Plasma and brain catecholamines in experimental uremia: acute and chronic studies. Life Sci..

[26] Siassi, F., Wang, M., Kopple, J. D. & Swendseid, M. E. Brain serotonin turnover in chronically uremic rats. *Am. J. Physiol*. **E526–8**; 10.1152/ajpendo.1977.232.5.E526 (1977). 10.1152/ajpendo.1977.232.5.E526 871160

[27] Graham, M. L. & Prescott, M. J. The multifactorial role of the 3Rs in shifting the harm-benefit analysis in animal models of disease. *Eur. J. Pharmacol*. 759, 10.1016/j.ejphar.2015.03.040 (2015). 10.1016/j.ejphar.2015.03.040 PMC4441106 25823812

[28] Kilkenny, C. *et al*. Improving Bioscience Research Reporting: The ARRIVE Guidelines for Reporting Animal Research. *PLoS Biol* **8**; 10.1371/journal.pbio.1000412 (2010). 10.1371/journal.pbio.1000412 PMC2893951 20613859

[29] Kim J. & Shin W. How to do random allocation (randomization). *Clin Orthop Surg*. **6**; 10.4055/cios.2014.6.1.103 (2014). 10.4055/cios.2014.6.1.103 PMC3942596 24605197

[30] Al Za’abi M, Ali N, Al Toubi M (2013). HPLC–fluorescence method for measurement of the uremic toxin indoxyl sulfate in plasma. J. Chromatogr. Sci..

[31] Zhang L, Yang JQ, Luo Y, Shang JC, Jiang XH (2016). Simultaneous determination of eleven compounds related to metabolism of bioamines in rat cortex and hippocampus by HPLC-ECD with boron-doped diamond working electrode. J. Pharm. Biomed. Anal..

[32] Pellow S, Chopin P, File SE, Briley M (1985). Validation of open:closed arm entries in an elevated plus-maze as a measure of anxiety in the rat. J. Neurosci. Methods..

[33] Boissier JR, Tardy J, Diverres JC (1960). Une nouvelle méthode simple pour explorer l’action’tranquillisante: le test de la chemine. Med. Exp..

[34] Buege JA, Aust SD (1978). Microsomal lipid peroxidation. Methods Enzymol..

